# Tools and data services registry: a community effort to document bioinformatics resources

**DOI:** 10.1093/nar/gkv1116

**Published:** 2015-11-03

**Authors:** Jon Ison, Kristoffer Rapacki, Hervé Ménager, Matúš Kalaš, Emil Rydza, Piotr Chmura, Christian Anthon, Niall Beard, Karel Berka, Dan Bolser, Tim Booth, Anthony Bretaudeau, Jan Brezovsky, Rita Casadio, Gianni Cesareni, Frederik Coppens, Michael Cornell, Gianmauro Cuccuru, Kristian Davidsen, Gianluca Della Vedova, Tunca Dogan, Olivia Doppelt-Azeroual, Laura Emery, Elisabeth Gasteiger, Thomas Gatter, Tatyana Goldberg, Marie Grosjean, Björn Grüning, Manuela Helmer-Citterich, Hans Ienasescu, Vassilios Ioannidis, Martin Closter Jespersen, Rafael Jimenez, Nick Juty, Peter Juvan, Maximilian Koch, Camille Laibe, Jing-Woei Li, Luana Licata, Fabien Mareuil, Ivan Mičetić, Rune Møllegaard Friborg, Sebastien Moretti, Chris Morris, Steffen Möller, Aleksandra Nenadic, Hedi Peterson, Giuseppe Profiti, Peter Rice, Paolo Romano, Paola Roncaglia, Rabie Saidi, Andrea Schafferhans, Veit Schwämmle, Callum Smith, Maria Maddalena Sperotto, Heinz Stockinger, Radka Svobodová Vařeková, Silvio C.E. Tosatto, Victor de la Torre, Paolo Uva, Allegra Via, Guy Yachdav, Federico Zambelli, Gert Vriend, Burkhard Rost, Helen Parkinson, Peter Løngreen, Søren Brunak

**Affiliations:** 1 Center for Biological Sequence Analysis Department of Systems Biology, Technical University of Denmark, Denmark; 2 Centre d'Informatique pour la Biologie, C3BI, Institut Pasteur, France; 3 Computational Biology Unit, Department of Informatics, University of Bergen, Norway; 4 Department of Veterinary Clinical and Animal Sciences, Faculty for Health and Medical Sciences, University of Copenhagen, Denmark; 5 School of Computer Science, University of Manchester, UK; 6 Department of Physical Chemistry, RCPTM, Faculty of Science, Palacky University, Czech Republic; 7 The European Bioinformatics Institute (EMBL-EBI), UK; 8 NEBC Wallingford, Centre for Ecology and Hydrology, UK; 9 INRA, UMR Institut de Génétique, Environnement et Protection des Plantes (IGEPP), BioInformatics Platform for Agroecosystems Arthropods (BIPAA), France; 10 INRIA, IRISA, GenOuest Core Facility, France; 11 Loschmidt Laboratories, Department of Experimental Biology and Research Centre for Toxic Compounds in the Environment RECETOX, Masaryk University, Czech Republic; 12 Bologna Biocomputing Group, University of Bologna, Italy; 13 Dept. of Biology, University of Rome Tor Vergata, Italy; 14 Department of Plant Systems Biology, VIB, Belgium; 15 Department of Plant Biotechnology and Bioinformatics, Ghent University, Belgium; 16 Faculty of Life Sciences, University of Manchester, UK; 17 Bioinformatics, CRS4, Italy; 18 Dept. of Computer Science, Systems and Communication. Univ. Milano-Bicocca, Italy; 19 UniProt, European Bioinformatics Institute (EMBL-EBI), UK; 20 SIB Swiss Institute of Bioinformatics, Switzerland; 21 Faculty of Technology and Center for Biotechnology, Universität Bielefeld, Germany; 22 Department of Informatics, Bioinformatics-I12, TUM, Germany; 23 Institut Français de Bioinformatique (French Institute of Bioinformatics), CNRS, UMS3601, France; 24 Albert-Ludwigs-Universität Freiburg, Fahnenbergplatz, 79085 Freiburg; 25 Centre for Molecular Bioinformatics, Dept. of Biology, University of Rome Tor Vergata, Italy; 26 Bioinformatics Centre, Department of Biology, University of Copenhagen, Denmark; 27 Centre for Functional Genomics and Biochips, Faculty of Medicine, University of Ljubljana, Slovenia; 28 Faculty of Medicine, The Chinese University of Hong Kong, China; 29 Hong Kong Bioinformatics Centre, School of Life Sciences,The Chinese University of Hong Kong, China; 30 Department of Biomedical Sciences, University of Padua, Italy; 31 Bioinformatics Research Centre (BiRC), Denmark; 32 Department of Ecology and Evolution, Biophore, Evolutionary Bioinformatics group, University of Lausanne, Switzerland; 33 STFC, Daresbury Laboratory, UK; 34 Department of Dermatology, University of Lübeck, Germany; 35 Institute for Biostatistics and Informatics in Medicine and Ageing Research, Rostock University Medical Center, Germany; 36 Institute of Computer Science, University of Tartu, Estonia; 37 Department of Computing, William Penney Laboratory, Imperial College London, UK; 38 IRCCS AOU San Martino IST, Italy; 39 Protein Research Group, Department for Biochemistry and Molecular Biology, University of Southern Denmark, Denmark; 40 Instruct, WTCHG, UK; 41 Central European Institute of Technology (CEITEC), Czech Republic; 42 National Bioinformatics Institute Unit (INB), Fundacion Centro Nacional de Investigaciones Oncologicas, Spain; 43 Dept. of Physics, Sapienza University, Italy; 44 Institute of Biomembranes and Bioenergetics, National Research Council (CNR), and Dept. of Biosciences, University of Milano, Italy; 45 Radboud University Medical Centre, CMBI, Netherlands; 46 Novo Nordisk Foundation Center for Protein Research, Faculty of Health and Medical Sciences, University of Copenhagen, Denmark

## Abstract

Life sciences are yielding huge data sets that underpin scientific discoveries fundamental to improvement in human health, agriculture and the environment. In support of these discoveries, a plethora of databases and tools are deployed, in technically complex and diverse implementations, across a spectrum of scientific disciplines. The corpus of documentation of these resources is fragmented across the Web, with much redundancy, and has lacked a common standard of information. The outcome is that scientists must often struggle to find, understand, compare and use the best resources for the task at hand.

Here we present a community-driven curation effort, supported by ELIXIR—the European infrastructure for biological information—that aspires to a comprehensive and consistent registry of information about bioinformatics resources. The sustainable upkeep of this Tools and Data Services Registry is assured by a curation effort driven by and tailored to local needs, and shared amongst a network of engaged partners.

As of November 2015, the registry includes 1785 resources, with depositions from 126 individual registrations including 52 institutional providers and 74 individuals. With community support, the registry can become a standard for dissemination of information about bioinformatics resources: we welcome everyone to join us in this common endeavour. The registry is freely available at https://bio.tools.

## MOTIVATION

Life sciences rely heavily on high-throughput technologies to understand, for example, the functional implications of gene structure, expression, regulation and variation upon human health, well-being and the environment. The outcome is an unprecedented huge volume of complex, highly heterogeneous biological information (1), which may span multiple scientific disciplines such as genetics, ecology and agriculture. In response, very many software tools and databases have been developed to manage and analyse the data. This presents a big challenge, not only for scientists, who must find relevant solutions in an ocean of possibilities, but also for ‘blue-collar bioinformaticians’ (as coined by Brad Chapman, http://bcb.io) who must solve a plethora of technical problems as they build usable protocols and workflows from technically diverse resources. It is therefore no surprise that bioinformatics help fora such as BioStar (2) are so popular.

There have been many efforts (including examples in the next section) that help guide people to find and use relevant bioinformatics software and databases. These include collections provided by individual academic institutes and research infrastructures, specialised formal registries and catalogues, software platforms, toolkits, system distributions, wikis, as well as multiple *ad hoc* lists on the Web. Although such initiatives serve their target audiences well, there is no single gateway to the available resources providing (i) consistency in the corpus of resource descriptions, (ii) adhesion to a common information standard and not least (iii) the foundation of a sustainable upkeep model that can obtain comprehensive coverage across the whole scientific and technical spectrum, and provide some assurance of quality in the long term.

We describe here a community-driven initiative, supported by ELIXIR, whereby multiple individuals from across the spectrum of bioinformatics, and involving users, developers and existing cataloguers of resources, have joined forces to build precisely such a registry from the bottom-up. The registry should help the efficient discovery and use of tools and thus provide a useful support for life science projects.

## COMMUNITY EFFORT

Bioinformatics is a ‘grass-roots’ industry, with many independent initiatives and a widespread sense of ownership of resources. Our approach follows from the belief that tool developers and service providers are best placed to document their own resources, and insofar as their enterprises are publicly funded, have a responsibility to share such information with others. Curation of any digital corpus to a high and consistent standard is, however, time consuming and costly. To ensure the registry is sustainable in the long term with limited resources, it is therefore essential, on one hand, to demonstrate incentives for contributors and, on the other, minimise future maintenance costs through decentralisation of the curation task. In short, we hope to leverage the “grass roots" via a coordinated curation effort where the workload is shared amongst many partners.

The strategy of aggregating or warehousing data via some community-driven, crowdsourced or federated effort is hardly new. Notable successes include Wikipedia (3), and many projects hosted on sites such as GitHub and SourceForge. Within bioinformatics, the strategy has been applied in diverse contexts, e.g. aggregation of database references within the UniProt database (4), the MIntAct (5) platform for curation of molecular interaction databases, data set sharing via BioMart (6,7), code sharing by the O|B|F Bio* libraries (8–15), user fora for questions and answers such as BioStar (2) and SEQanswers (16) and sharing of software skills via Software Carpentry (17). Various initiatives include cataloguing of bioinformatics resources, such as the Molecular Biology Database List published in the *Nucleic Acids Research*'s Database Issues (18–20), the Bioinformatics Links Directory (21,22), the EMBRACE Registry (23,24) and BioCatalogue (25) of Web services; software sharing and distribution initiatives such as Bioconductor (26), Bio-Linux (27), Debian Med (28), or BioJS (29–31); and the tools sections at BioStar and SEQanswers—SEQWiki (32). Indeed, all these have fundamentally depended upon wide community contributions.

We propose and have implemented a sustainable ‘federated curation model’ for bioinformatics tools and data resources whereby developers, providers, integrators and cataloguers maintain and share information about the resources within their scope: curation responsibilities are thus distributed. The registry collates and serves a unified ‘snapshot’ of the available information distributed on the Web, and provides support and tools for annotation of resources to a common standard. By aggregating content from external sources, we leverage existing communities and the valuable documentation that has already been created. Contributors not only provide content, but also help develop the underlying ontology, EDAM (33), used for semantic annotation of the registered resources, via the mechanisms described below.

## REGISTRY CURATION AND DEVELOPMENT

**Figure 1.**
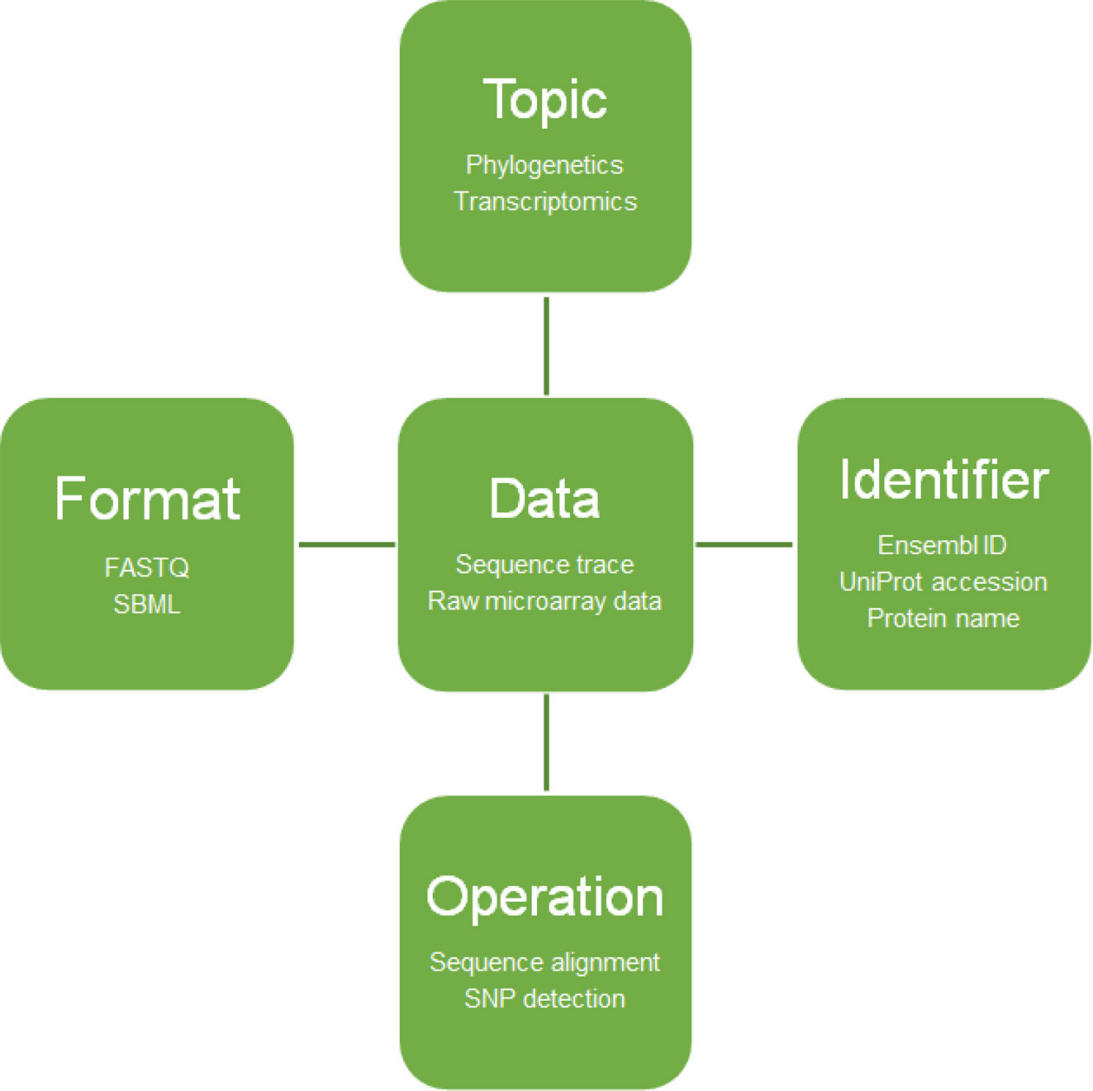
EDAM concepts. EDAM includes four main sub-ontologies defining common concepts within bioinformatics: topics, operations, data (including identifiers, the fifth sub-ontology) and data formats. EDAM provides the core scientific concepts for describing registry entries.

In practical terms, registry curation involves the annotation of resources to bring their description up to a mandated minimum standard of information, the registration of those descriptions, subsequent updates of accessions and concomitant ontology development. The information standard is defined by biotoolsXSD^1^, a formalised XML schema (XSD) of key scientific, technical and administrative attributes, including scientific concepts from the EDAM ontology (Figure 1). EDAM provides the core vocabulary of well established, familiar concepts that are prevalent within bioinformatics, including types of data and data identifiers, data formats, operations and topics. The remaining required controlled vocabularies, for example for resource type and software licences, are defined internally within biotoolsXSD. The schema defines a total of 55 fields of information of which 10 are mandatory (Table 1).

**Table 1.** Mandatory resource information. Of the 55 fields of information defined in biotoolsXSD—the resource description model used by the registry, 10 are mandatory and provide a minimum standard for annotation of registered resources

Field	Description	Format
Name	The canonical name of the resource	Text
Homepage	Resource homepage	URL
Description (1 only)	Short textual description of the resource	Text
Resource type (1 or more)	Basic resource type	‘Database’, ‘Tool’, ‘Service’, ‘Workflow’, ‘Platform’, ‘Container’, ‘Library’ or ‘Other’
Interface type (1 or more)	Resource interface type	‘Command-line’, ‘Web UI’, ‘Desktop GUI’, ‘SOAP WS’, ‘HTTP WS’, ‘API’ or ‘QL’
Topics (1 or more)	General scientific domain(s) the software serves, e.g. ‘Proteomics’.	URI of EDAM Topic
Functions (1 or more)	Functions (1 or more), e.g. ‘Gene regulatory network prediction’	URI of EDAM Operation
Input types (0 or more)	Type of data : primary input(s), e.g. ‘Protein sequences’	URI of EDAM Data
Output types (0 or more)	Type of data : primary output(s), e.g. ‘Protein sequence alignment’	URI of EDAM Data
Contact (1 or more)	Primary contact, e.g. a person, helpdesk, or mailing list	Email or URL

Registration mechanisms have been tailored to the needs of contributors, ranging from lone developers of one of a few tools, to large institutional providers or other registries that store information on hundreds. The mechanisms currently include a Web-based interface for manual creation and editing of resource descriptions, an HTTP-based API for automated creation and update of accessions, and a Google Sheets format for spreadsheet-style editing. These methods may be used in combination with one another during the upkeep of accessions, with curation assistance and quality checks provided centrally by ELIXIR: the registry team will support contributors in the important task of content upkeep, including helping to identify and remove stale entries, update and improve existing annotations, as well as provide new content.

The strategy for registry growth relies upon an active network of curators, coordinated by ELIXIR, and adheres to certain principles such as those enumerated by Aidan Budd *et al*. (34) that provide the foundation for a successful bioinformatics community. These principles are manifest by providing a coherent vision and organisation, and by organising participatory activities which facilitate work and communication in a productive and appealing environment. The activities have included scoping of requirements, surveys, interviews and —crucially—a range of community-led events including various hackathons. A total of 15 events thus far have included Debian Med Sprints, BOSC Codefests (35,36) and various workshops organised by ELIXIR and BioMedBridges. These events broadly follow the guidelines as elaborated by Budd *et al*. in (37), and are of four types:

- **General Hackathons** are akin to Codefests, but for documentation. They gather providers from across the board to curate their resources, critique the registry and EDAM, develop applications and provide a forum for knowledge exchange and collaboration.
- **Thematic Hackathons** engage experts in a specific scientific area to help improve the documentation of resources within the theme, improve the relevant branches of EDAM, consolidate the existing registry annotations, as well as register new resources.
- **Resource Hackathons** collaborate with representatives of a specific collection of tools and services, typically some other registry, community project or Web portal, to bring the collection up to the information standard and expose it in the registry.
- **Technical Hackathons** focus on ontology, software or other technical developments in support of curation of the registry, its technical development, applications and integration with other systems.

The events, which have engaged individuals, projects and institutes within and beyond ELIXIR, have proved to be an efficient way to enhance and expand the content of the registry and EDAM (see below), while providing resource descriptions that are applicable for community use. Further, they have addressed various tasks, including:

- prioritisation of attributes in the resource description model
- critique of EDAM and the definition of new concepts
- critique of the registry interfaces, including usability tests, user persona evaluation, *etc*.
- registry software feature requests and prioritisation of requirements using agile methodology
- prioritisation of registry curation and scientific themes
- harvesting suggestions for registry upkeep strategy, applications and collaborations

These outputs cement the effort in the user community and ensure that all key registry developments are user-driven. We expect to organise approximately half a dozen events per year in future and are open to suggestions and participation.

## REGISTRY CONTENT

The registry content currently (November 2015) includes 1785 resources (Table 2), with depositions from 126 individual registrations, including 1714 resources from 52 institutional providers and 71 resources from 74 individuals. Contributions have been received or are pending from a broad range of institutes and projects (Table 3) and represent a cross-section of the types of providers, integrators and cataloguers of bioinformatics resources, who we anticipate will continue contributing to future growth.

**Table 2.** Content of the Tools and Data Services Registry. The registry includes 1633 accessions with a total of 36,428 annotations. The table gives a breakdown of the types of resources and their interfaces, and the number of scientific annotations made using EDAM

	#entries or #annotations
#entries, with breakdown by resource type	*Total (1785)*, Tool (1396), Database (306), Library (67), Platform (31), Service (6), Workflow (2), Container (1), Other (10)
#annotations, with breakdown by type	*Total (48 105)*, EDAM annotations (11 093), other controlled vocabularies (7850), URLs (7076), Text/IDs (22 086)
#entries, with breakdown by interface type	*Total (1785)*, Command line (780), Web UI (769), SOAP WS (163), HTTP WS (135), Desktop GUI (31), API (27), QL (2)
#EDAM annotations, with breakdown by type	*Total (11 093)*, Data (3599), Function (2602), Topic (2681), Format (2211)

**Table 3.** Resource providers. A non-exhaustive list of collections that have contributed or will contribute to the registry. The list includes a cross-section of bioinformatics service providers including other catalogues such as SEQwiki and BioCatalogue

Name, URL, Short description
**CBS Prediction Servers**
http://www.cbs.dtu.dk/biotools
A collection of on-line prediction services from CBS-DTU. The resource contains 75 tools for gene finding and splice sites, post-translational protein modification, immunological features, protein function and structure, protein sorting, genomic epidemiology and more. The tools can be used via interactive input forms, with many available as software packages and SOAP Web services.
**DRCAT resource catalogue**
http://drcat.sourceforge.net
The data resource catalogue is a collection of metadata on bioinformatics Web-based data resources. The catalog contains over 600 resources including bioinformatics and biomedical databases, ontologies, taxonomies and catalogues.
**BiBiServ**
http://bibiserv.cebitec.uni-bielefeld.de
BiBiServ is a collection of bioinformatics tools that emerged from the research at Bielefeld University. It contains over 40 mainly analysis and utility tools, including RNA structure prediction, metagenomics, genome rearrangement, alignments, evolutionary relationships, primer design and suffix trees. These are available as interactive web applications, HTTP Web services and downloadable software.
**BINF.KU.DK Services and Software**
http://www.binf.ku.dk/services
A collection of over 20 web services, databases and software packages from The Bioinformatics Centre at The University of Copenhagen. The resource covers sequence and structure analysis, prediction and modeling, gene regulation, population genetics and more.
ELIXIR-CZ Services collection
https://www.elixir-czech.cz/services
The Czech Bioinformatics Services resource is provided by members of ELIXIR CZ node. It contains over 30 bioinformatics tools and databases for analysis of sequence, topology and structure of nucleic acids and proteins to genomics, proteomics and benchmarks for small molecule interactions. The databases can be accessed via web GUIs while tools are available as web, standalone and command-line applications.
**ELIXIR eLearning Platform**
https://elixir.mf.uni-lj.si
**Orange**
http://orange.biolab.si/
Orange data mining suite is an open source data visualization and analysis software for data mining through visual programming or Python scripting. It consists of over 100 components for machine learning and add-ons for bioinformatics and text mining.
**GoMapMan**
http://www.gomapman.org
GoMapMan is a database of gene functional annotations in the plant sciences based on the plant-specific MapMan ontology.
**ELIXIR-ITA Services collection**
A collection of services provided by research institutions members of the ELIXIR Italian node. The resource includes databases and analysis tools developed and maintained by Italian bioinformatics groups and institutions.
**University of Padova ELIXIR-ITA-PADOVA**
http://protein.bio.unipd.it/
A collection of 60 bioinformatics tools from the University of Padova.It includes databases for structural bioinformatics and genome sequences as well as tools for sequence analysis, phylogenetics, structure analysis, chemioinformatics and network analysis.
**Bologna Biocomputing group predictors and services**
http://biocomp.unibo.it/predictors.html
A collection of 22 predictors for subcellular localization, disease-related mutations and protein sequences annotation from Bologna Biocomputing Group. Most tools are accessible using a Web UI while some offer a command line interface.
**Sapienza University Biocomputing group resources and tools**
http://www.biocomputing.it/index.php/About-us/tools
A collection of 19 resources and tools for structural bioinformatics, immunoinformatics and genomics from the Sapienza University Biocomputing Group.
**Molecular Genetics Group ELIXIR-ITA-TORVERGATA**
http://moleculargenetics.uniroma2.it
A collection of 7 databases and portals linking physically and functionally gene products. All databases and portals data can be searched, visualized and downloaded through Web UI interfaces.
**Molecular Bioinformatics Group ELIXIR-ITA-TORVERGATA**
A collection of tools dedicated to the analysis of protein structures, the identification of structure motifs and the comparison of RNA secondary structure.
**Milano-Bicocca Resources and Tools ELIXIR-ITA-BICOCCA**
Online services and open source software, mainly for NGS and EST analysis or to infer evolutionary histories in tumors. The majority of tools are used via a command line interface while the rest offer a graphical interface.
**Mobyle@Pasteur**
http://mobyle.pasteur.fr
A collection of 300 bioinformatics tools covering various topics such as sequence analysis, phylogeny, integrated in an online workbench. The suite is a combination of tools developed at the Institut Pasteur and/or tools used by it, for research and education.
**Galaxy@Institut Pasteur**
https://galaxy.web.pasteur.fr
A collection of 260 bioinformatics tools, mainly dedicated to NGS analysis, and integrated into the Galaxy instance available at the Institut Pasteur. This instance is only available to Pasteur researchers and collaborators.
**GenOuest**
http://www.genouest.org
A collection of tools dedicated to the analyses of NGS data along with bioinformatics genomic databases hosted by GenOuest. Most tools can be used via command line, while the databases and some of the tools are available through a web interface.
**IFB ELIXIR-FR**
http://www.france-bioinformatique.fr/?q=en/services
The French Institute of Bioinformatics (IFB) is a national service infrastructure in bioinformatics that gathers together the bioinformatics platforms of the main French research organizations, CNRS, INRA, INRIA, CEA and INSERM, as well as CIRAD, the Pasteur and Curie Institutes and the French universities. IFB's principal mission is to provide basic services and resources in bioinformatics for scientists and engineers working in the life sciences. IFB is the French node of ELIXIR.
**Loschmidt laboratories software resources**
http://loschmidt.chemi.muni.cz/peg/software/
A collection of tools developed at Loschmidt Laboratories for protein design, engineering and analysis. The tools are mostly available via web interface or as command line application.
**Identifiers.org** **Registry**
http://identifiers.org/registry/
The core of the Identifiers.org Registry is a catalogue of data collections—corresponding to controlled vocabularies, databases and more—along with their URIs and the associated physical URLs.
**INB Services**
http://www.inab.org/resources/list-of-all-systems-and-tools/
The catalog of Spanish National Bioinformatics Institute. INB Services develops and provides software tools and web servers for the global life sciences research community.
**PSB resources**
http://bioinformatics.psb.ugent.be/software
A collection of tools developed at the department of Plant Systems Biology (VIB,Gent University). The tools cover topics such as comparative genomics, network analysis, genome prediction, annotation and visualization. The tools are available as web UI or command line applications.
**CRS4 resources**
http://orione.crs4.it
Biocomputing infrastructure to primarily support analysis of data produced by the CRS4 NGS facility. The system integrates hundreds of tools into a web-based traceability framework that can handle the whole transformation process from raw data to downstream analysis.
**CCP4**
http://www.ccp4.ac.uk
A collection of computational tools for macromolecular X-ray crystallography, and other biophysical techniques.
**Instruct**
https://www.structuralbiology.eu/update/toolbox
A collection of computational tools for structural biology from Instruct. Instruct is a pan-European research infrastructure in structural biology, making high-end technologies and methods available to users.
**University of Tartu bioinformatics resources**
http://biit.cs.ut.ee, http://bioinfo.ut.ee
Estonian bioinformatics services, tools and databases provided by ELIXIR-Estonia contain almost 20 tools and databases for several high-throughput analyses, enrichment analysis, network dissection, primer design approaches, as well as data visualisation applications. The resources are mainly available as interactive web applications and R packages.
**ExPASy/SIB resources**
http://www.expasy.org
ExPASy is the SIB bioinformatics resources portal which provides access to scientific databases and software tools (i.e. resources) in different areas of life sciences including proteomics, genomics, phylogeny, systems biology, population genetics, transcriptomics.
**SEQanswers wiki**
http://seqanswers.com/wiki/Software
The SEQanswers wiki (SEQwiki) is a wiki database that is actively edited and updated by the members of the SEQanswers community (http://seqanswers.com). The wiki provides an extensive catalogue of tools, technologies and tutorials for high-throughput sequencing.
**USMI Cell Line Databases and Analysis Tools**
http://bioinformatics.hsanmartino.it
The resource is devoted to management and distribution on information on human and animal cell lines and other biological resources. The tools are usually available as a web interface or as REST and SOAP Web Services.
**BioCatalogue**
http://biocatalogue.org
The BioCatalogue is a curated catalogue of 369 life science Web Services. Users and curators register metadata about Web Services. Web Services in the catalogue can be either SOAP or REST APIs.
**SDU bioinformatics tools collection**
http://www.sdu.dk/en/Om_SDU/Institutter_centre/Bmb_biokemi_og_molekylaer_biologi/Forskning/Forskningsgrupper/Protein/Bioinformatics or https://elixir-registry.cbs.dtu.dk/?q=bmb.sdu.dk
Collection of tools and services developed and maintained at the University of Southern Denmark currently comprising 13 applications. Covered topics are cluster validation, proteomics, pathway and network processing, and omics analyses.
**University of Bergen and ELIXIR-NO tools**
http://www.bioinfo.no/applications
A list of over 30 tools, including web applications and Web services, provided by the universities in Norway affiliated with ELIXIR-NO.
**Tools@EBI**
http://www.ebi.ac.uk/services
A portfolio of bioinformatics tools to facilitate scientific discovery within the life sciences, provided by EMBL-EBI.
**GO tools registry**
http://geneontology.org
A collection of resources to perform data analysis using Gene Ontology (GO). Includes tools developed by GO Consortium members as well as some third-party resources.
**EMBOSS**
http://emboss.sf.net
EMBOSS is a free Open Source software analysis package specially developed for the needs of the molecular biology (e.g. EMBnet) user community. The software automatically copes with data in a variety of formats and even allows transparent retrieval of sequence data from the web. Also, as extensive libraries are provided with the package, it is a platform to allow other scientists to develop and release software in true open source spirit. EMBOSS also integrates a range of currently available packages and tools for sequence analysis into a seamless whole. EMBOSS breaks the historical trend towards commercial software packages.
**WHAT-IF**
http://swift.cmbi.ru.nl/whatif
A versatile molecular modelling package that is specialized on working with proteins and the molecules in their environment like water, ligands, nucleic acids, etc.
**Bio-Linux**
http://environmentalomics.org/bio-linux
Bio-Linux is an Ubuntu Linux-based distribution that adds more than 250 bioinformatics packages, providing around 50 graphical applications and several hundred command line tools, as well as the Galaxy environment for browser-based data analysis and workflow construction.
**Debian Med**
https://www.debian.org/devel/debian-med/
Debian Med is a project that aims at developing Debian into an operating system that is particularly well fit for the requirements for medical and biological research. The goal of Debian Med is a complete free and open-source system for all tasks in life-scientific research. To achieve this goal Debian Med integrates applicable software into Debian.
**Rostlab**
http://rostlab.org
A collection of bioinformatics tools for the prediction and analysis of the aspects of protein structure and function, provided by the Rost lab at the Technical University of Munich and Columbia University of New York.
**BioJS**
http://biojs.net
A registry and open-source library of JavaScript components to visualise biological data.

A total of 48 105 annotations (information fields) have been completed on the entries of which 11 093 are EDAM annotations (Table 2). The rest are annotations from controlled vocabularies (7850)—for example for licenses and interface types—defined within biotoolsXSD, URLs (7076), or short textual descriptions and IDs such as DOIs (22 086). The content includes mostly tools and a significant number of databases, most of which have a Web GUI, with a significant proportion having a command-line interface, or a programmatic API via HTTP or SOAP Web services.

**Figure 2.**
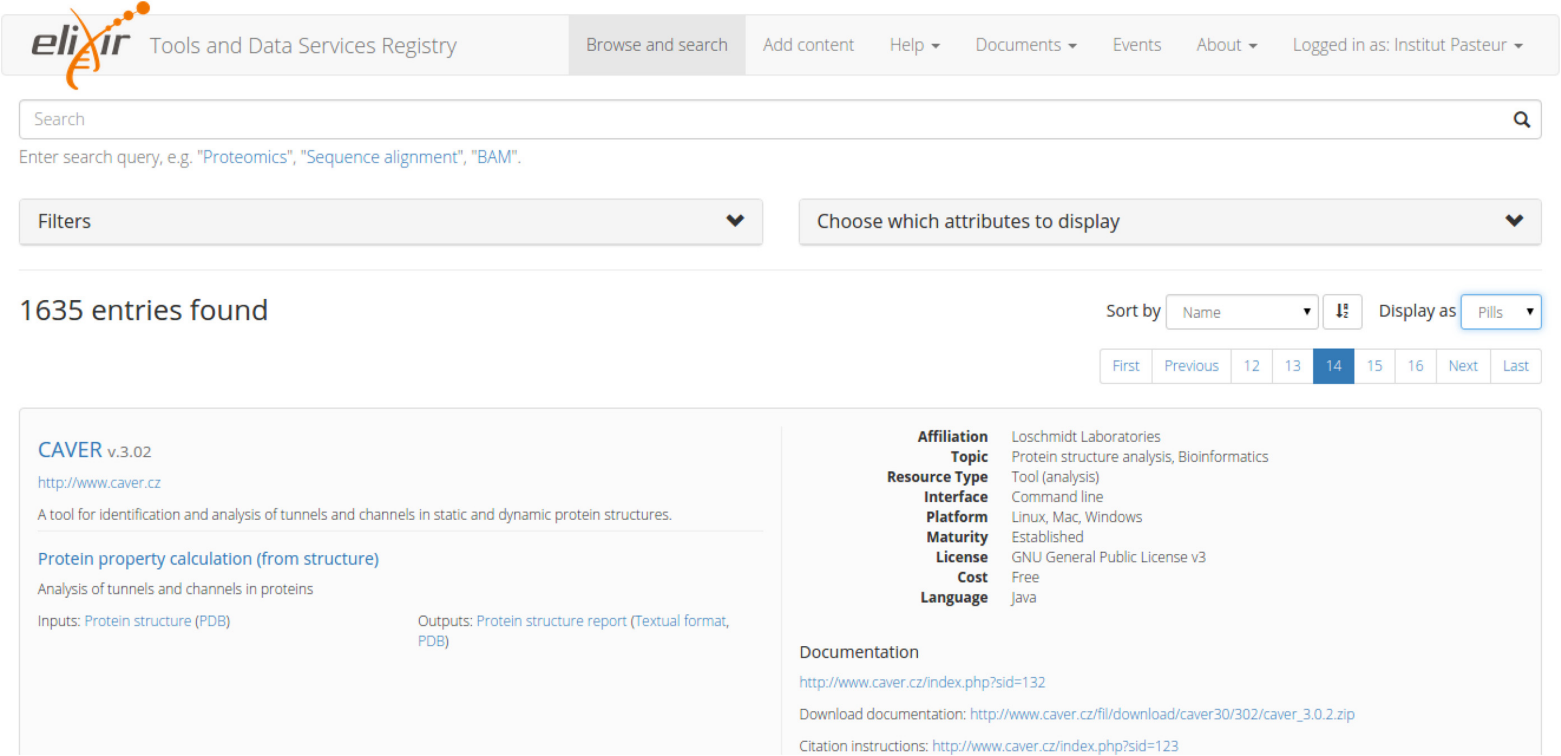
ELIXIR registry query user interface. The query interface (https://bio.tools) provides features to search the registry, display what fields of information are shown, and filter and sort the results by various attributes.

The registry content is made available for browsing and searching via an interactive query interface (Figure 2). The interface provides features to search the corpus of resource descriptions, display what fields of information are shown and filter and sort the results by various attributes. Thus, a user may formulate a precise query, that addresses a specific bioinformatics task, and quickly retrieve resources that fulfil those exact requirements. The search results are available for viewing in a spreadsheet-like view (‘grid’) and in a summary form (‘pills’). A URL-based API supports programmatic queries.

A secondary but important result is the community development of EDAM that has occurred in support of the registry growth. Since the inception of the registry, there have been a total of eight new EDAM releases, mostly in follow-up to registry events and through collaborations with contributors. The changes include addition of new concepts and synonyms, but also some structural changes to improve the usability of EDAM. All EDAM development is use-case drive. The registry is thus currently the primary driving force in EDAM's development.

The registry content is available under the Creative Commons Attribution licence (CC BY 4.0). The registry code itself is licensed under the GNU General Public License (GPLv3). biotoolsXSD (and in future other community-developed components) are freely available^2^.

## DISCUSSION

We have described here a registry whose content depends upon a community effort that aspires to provide for bioinformatics resources, at least a minimum documentation conforming to consistent semantic and syntactic standards. The work represents the first step towards a comprehensive registry, the further development of which should bring progressive benefits: scientists using the registry to find, understand, compare and select resources should benefit from a process that yields relevant results more efficiently than, say, trawling the Web. Developers and service providers contributing to the registry should benefit by increased exposure of their resources which in turn yields more usage, more visibility and citations, as well as bug reports and suggestions for new features and improvements.

Our approach has several advantages. Firstly, the distributed nature and emphasis on community activities means that, rather than duplicating curation efforts, curation is driven by and tailored to local needs, and should therefore be sustainable in the long-term. Secondly, the same community is contributing to the standards for resource description, providing all-important scientific relevance and consistency. Finally, the aggregation of diverse types of tools and data resources should help the ‘blue-collar bioinformatician’ in the management of their day-to-day workflows. Many of the previously cited catalogue efforts have been specific to a particular kind of tools, and therefore did not provide the ‘one stop shop’ that would be so helpful in this regard.

Success is predicated ultimately upon the goodwill of enthusiastic individuals, backed up by institutional support, to assume responsibility for the resources within their purview. Thus, the pressing requirement is to build and support the community behind the registry, but we have strong grounds to expect this effort will succeed in the long-term. Firstly, there are natural incentives to contribute to a common effort in which the curation burden is shared. Secondly, the approach shares a similar philosophy to other community projects such as DebianMed and SEQwiki, making it easy to find like-minded people to work with productively. Finally, the anchoring of the effort within ELIXIR provides a global context and some resources to develop the registry. The Danish node of ELIXIR—the ‘tools node’—is coordinating and fostering the effort, and will leverage relevant initiatives such as the ISB International Society of Biocuration. Hence we follow a dual approach, addressing the problem both from the ‘bottom-up’ and the ‘top-down’, as elucidated in Budd *et al*. (37).

From the outset, an agile user-centered approach has been taken to the registry technical development, scientific content, upkeep strategy and social aspects. This will continue, and ensure that the needs and desires of content providers and end-users are satisfied. Growth in the curation network will extend and improve the content, and registry functionality, in an organic way. We anticipate this will include new types of resources, for example those based on virtual, cloud or container-based infrastructure, such as Docker, in addition to essential services defined by ELIXIR partners and community projects. To support this growth, improved tooling for community curation of the registry and EDAM will be developed. Once the content expands to provide a clearer picture of which tools are re-used or provided in various contexts, we shall define a core ‘reference set’ of tool descriptions, validated and annotated to a very high standard and available for re-use by others. This set will be referred to within the registry by any collections or services that include or provide that tool, mitigating redundancy of both the registry content and the curation effort. Beyond these basic developments, various applications will be pursued, including:

- Further development of ReGaTE ^3^, a tool that automates the registration of Galaxy instances in the ELIXIR registry
- Interoperability with workbench systems, to facilitate integration of resource descriptions into workbench environments (38)
- Crosslinking and integration with other systems and initiatives planned within ELIXIR, including the benchmarking and monitoring of tools, the TeSS training portal and the eLearning platform ^4^

With support, the registry can become a community standard for the dissemination of information about bioinformatics resources. We actively encourage others to integrate the registry content and EDAM into their own portals, develop applications and contribute to the emerging common curation effort. There are various practical ways to get involved, including getting a registry account and registering your resources, participating at dedicated hackathons, joining the mailing lists, contributing to EDAM, spreading the word and of course documenting the resources you provide or use, for example, at your local site, or by helping out with Debian package annotation, editing SEQwiki and so on. We welcome everyone concerned with the provision or use of bioinformatics resources to join the common endeavour, coordinated by ELIXIR but open to everyone within the life sciences.

## Footnotes

1. Available at https://github.com/bio-tools/biotoolsxsd

2. See at https://github.com/bio-tools/

3. See http://regate.readthedocs.org/en/latest/

4. See https://elixir.mf.uni-lj.si/
